# Combined Robotic VErticalization and Lower Limb Mobilization in Patients with Severe Acquired Brain Injury: Protocol of a Multicenter Randomized Controlled Trial (VEM-sABI) †

**DOI:** 10.3390/jcm14186628

**Published:** 2025-09-20

**Authors:** Anna Estraneo, Maria Rosaria Fiorentino, Alfonso Magliacano, Maria Assunta Puopolo, Ilaria Rivetti, Maria Cristina Messa

**Affiliations:** 1 IRCCS Fondazione Don Carlo Gnocchi ONLUS, 50143 Florence, Italy; mafiorentino@dongnocchi.it (M.R.F.); amagliacano@dongnocchi.it (A.M.); mpuopolo@dongnocchi.it (M.A.P.); irivetti@dongnocchi.it (I.R.); 2 IRCCS Fondazione Don Carlo Gnocchi ONLUS, 20148 Milan, Italy; cristina.messa@unimib.it; 3 School of Medicine and Surgery, University of Milano-Bicocca, 20126 Milan, Italy

**Keywords:** acquired severe brain injury, verticalization, rehabilitation, severe brain injury, disorder of consciousness, robotics

## Abstract

**Background**: Upright position recovery (i.e., verticalization) is crucial in the rehabilitation of severe acquired brain injury (sABI). VErticalization by tilt table equipped with robotic-assisted lower limbs cyclic Mobilization (VEM) may facilitate a safer adaptation to vertical posture, reducing orthostatic hypotension occurrence. This multicenter randomized controlled trial (RCT) aims at investigating efficacy, safety, and usability of VEM compared to Traditional Verticalization (TV) using a conventional tilt table in cognitive-motor rehabilitation of sABI patients; **Methods**: a total of 118 sABI patients with or emerged from prolonged Disorder of Consciousness (pDoC and eDoC) will be enrolled in six post-acute Neurorehabilitation Units and randomly allocated to VEM or TV arm (for each arm: total 25 sessions of 30 min daily treatment/5 days/week/5 weeks). Patients will undergo clinical–functional assessment, resting EEG recording and blood sampling, before, at the end of treatment, and after 1 month; **Results**: we will expect possible differences in safety and usability of verticalization between VEM and TV rehabilitative intervention and in their efficacy to improve clinical–functional findings and brain indices; **Conclusions**: this RCT will provide new insights for the intensive, tailored and safe neurorehabilitation intervention in patients with sABI.

## 1. Introduction

Survivors from a severe Acquired Brain Injury (sABI; i.e., severe traumatic or non-traumatic brain injury leading to a comatose state with a Glasgow Coma Scale ≤ 8 for at least 24 h in the acute phase) can persist in a clinical condition of prolonged Disorder of Consciousness (pDoC; i.e., patients in vegetative state/unresponsive wakefulness syndrome, VS/UWS, or in minimally conscious state, MCS) or emerge from DoC (eDoC) with a moderate or severe cognitive motor disability [1]. Both American and Italian guidelines recommended an early post-acute admission of such complex disabled individuals in specialized rehabilitation units with high expertise in multidisciplinary care and multimodal diagnosis and prognosis [1,2]. These dedicated settings provide a comprehensive rehabilitation program that includes medical stabilization, alternate positioning, motor and cognitive training and speech therapy, to prevent and treat clinical complications (e.g., contractures, spasticity, and heterotopic ossifications) and to facilitate recovery of cognitive-motor and functional abilities.

In the rehabilitation phase, the early recovery of the upright position (i.e., verticalization) is crucial to prevent secondary complications due to immobility (e.g., stasis pneumonias, deep vein thrombosis, pressure ulcers) [3], enable the stabilization of the hemodynamic balance [4], promote the cognitive performance improvement (especially vigilance and attention) [5], and to ameliorate the muscular-cutaneous trophism and the control of trunk and limbs. However, the Traditional Verticalization (TV) using a conventional tilt table can be hampered by orthostatic hypotension due to cardiac output reduction because of blood pooling in the lower extremities and/or co-existing central sympathetic system impairment [6]. Since its introduction by Czell in 2004 [7] the tilt table equipped with robotic-assisted lower limbs cyclic mobilization has been proposed as a safe and suitable device for a controlled verticalization to facilitate the adaptation to vertical posture in bedridden patients with brain-injury since the acute phase [8]. A combined assisted lower limbs mobilization can activate the musculo-venous pump that enhances counter gradient venous blood flow return to the right heart from systemic circulation, thus resulting in better cardiac output and lower side effects (e.g., orthostatic hypotension) occurrence [9,10]. However, despite the potential benefits and growing popularity of robot-based rehabilitation [11], no conclusive data on cognitive-motor effects of controlled verticalization in patients with sABI have been found, likely because of the methodological bias of the available clinical trials (e.g., small or heterogeneous cohorts ranging from acute to chronic patients, absence of clear randomization; lack of blinding in outcome assessors [5,8,12,13,14]. To minimize bias due to confounding, the present randomized controlled trial (VEM-sABI) will compare multiple clinical, neurophysiological, and biological effects of combined robot-assisted VErticalization and lower limbs Mobilization (VEM) versus a TV in a large cohort of patients with sABI admitted to multiple post-acute rehabilitation settings. The primary aim of the VEM-sABI study will be to evaluate the efficacy of VEM versus TV in promoting short-term recovery of cognitive functioning in a large cohort of patients with sABI. Secondary aims will be to evaluate VEM vs. TV in: (i) preventing or reducing spasticity; (ii) promoting lower limb movements; (iii) reducing functional disability; (iv) and an additional secondary aim will be to evaluate the usability and safety of the two devices from the professional’s point of view. Exploratory aims will be to evaluate the efficacy of VEM vs. TV in: (i) promoting the recovery of consciousness in patients with pDoC; (ii) preventing clinical complications; (iii) maintaining the possible improvement in clinical–functional measures at 1 month after treatment. Additionally, it is crucial to also investigate the patients’ physiological features (such as neurophysiological indices of brain activity and biomarkers of brain damage and plasticity) to accurately outline patients with higher probability to respond to the treatment and detect sub-clinical outcomes [15]. In this context, additional exploratory objectives of study will be to evaluate the effect of VEM vs. TV on brain functionality and neuronal plasticity.

## 2. Materials and Methods

### 2.1. Trial Design and Participating Centers

This is a non-commercial, non-profit clinical investigation conducted under the “Fit for Medical Robotics” initiative, funded by the Italian Ministry of University and Research via the National Complementary Plan to the National Recovery and Resilience Plan, Investment I.1 (Call D.D. 931/2022).

This multicenter study is a single-blinded, randomized controlled trial (RCT), with two treatment-arms (1:1; VEM and TV), enrolling patients with sABI admitted to 6 Italian post-acute neurorehabilitation units (see Acknowledgments). The neurorehabilitation units were selected for their high expertise in the care of patients with sABI. Two additional expert laboratories will analyze blood biomarkers and will build and manage the electronic database and run a quantitative analysis of EEG (see Acknowledgments for the contributors of participating centers). The study protocol has been elaborated according to the Standard Protocol Items: Recommendations for Interventional Trials (SPIRIT; see Table 1) [16,17] and the Template for Intervention Description and Replication (TIDieR) checklist (see Supplementary Materials). The study was registered at ClinicalTrials.gov (ID: NCT06469983) on 14 June 2024.

### 2.2. Study Population

All patients with sABI consecutively admitted to the participating centers who meet the inclusion and exclusion criteria reported in Table 2 will be enrolled. Eligible conscious patients or legal representative/primary caregiver of pDoC patients will be orally informed about the rationale, aims, and procedures of the study in order to obtain their written informed consent. Purposes, procedures, and time points of the study will be clearly explained after a semi-formalized interview, whose structure is shared across centers, to either the eligible patients (if the Level of Cognitive Functioning, LCF, is between 7 and 8) or the patient’s legal representative/primary caregiver (if the assessment of cognitive functioning suggests that the patient may not understand all the information, e.g., for patients with LCF between 2 and 6). In the latter case, if a patient were to reach a higher LCF during treatment, she/he would be personally asked to sign an informed consent form.

#### Sample Size

Sample size was estimated based on information from a previous study [18] enrolling patients with MCS allocated to advanced robotic verticalization using the Erigo^®^ (Hocoma, Volketswil, Switzerland) device or traditional verticalization with a traditional tilt table. The comparison of the LCF score between the two groups after eight weeks of treatment led to an increase from 3.13 ± 1.41 to 3.60 ± 1.50 in the control group (n = 15) and from 3.60 ± 1.05 to 4.40 ± 1.06 in the experimental group (n = 15; effect size f = 0.248). For a two-sided α of 0.05 and a power of 80%, we will estimate enrolling at least 49 subjects per arm (VEM and TV). To account for a possible dropout rate of 20%, a total of 118 patients will be enrolled (59 subjects per arm).

### 2.3. Randomization and Trial Intervention

#### 2.3.1. Randomization

**Figure 1:**
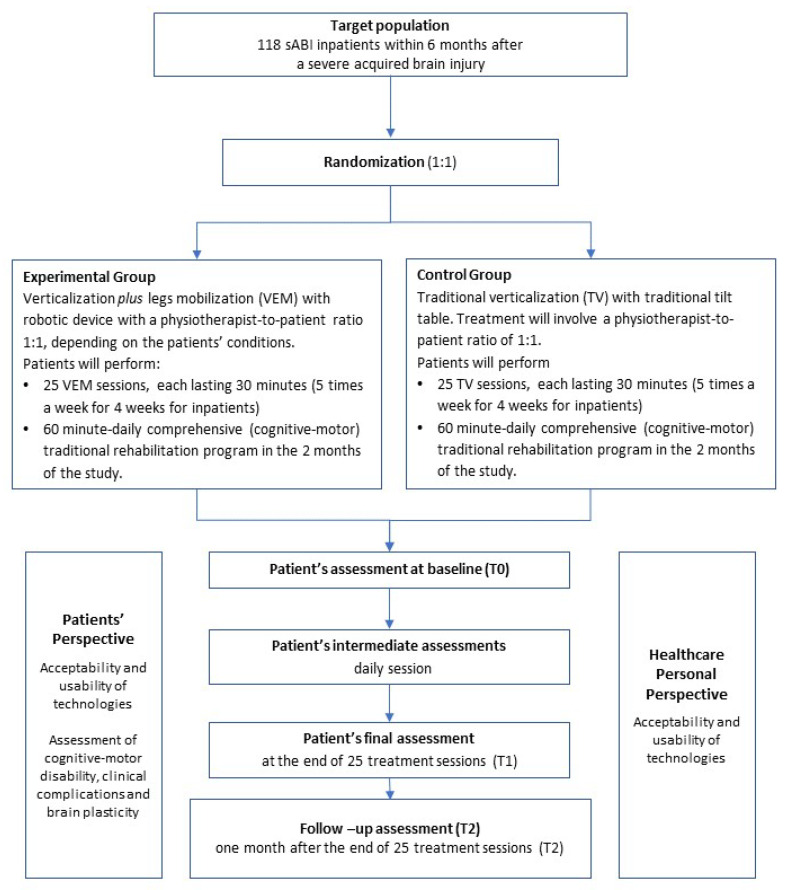
CONSORT flow diagram. Abbreviations: sABI = severe Acquired Brain Injury; TV = Traditional Verticalization; VEM = robotic Verticalization and Mobilization.

The eligible patients will be randomly allocated to either experimental (VEM) or control (TV) arm in a 1:1 ratio stratified by recruiting center (see Figure 1 for the CONSORT flow diagram). The sequence, generated by the Sealed Envelope randomizer is then uploaded in the Research Electronic Data Capture (REDCap) database [19]. Clinicians, physiotherapists, and patients will not be blinded with respect to the interventions because of the intervention’s nature. However, the clinical–functional, neurophysiological, and biological assessments will be performed by examiners who will be blinded to the patient’s treatment group. The statistician in charge of the analysis will also remain blind.

#### 2.3.2. Trial Protocol

After screening for inclusion/exclusion criteria, a patient is enrolled in the trial and the following demographic and anamnestic variables will be collected: age, sex, education; medical history: time post-injury (TPI), etiology, morphometry and location of brain injury by brain CT scan; pharmacological therapy affecting arousal (e.g., neurosedatives); clinical diagnosis of pDoC (i.e., VS/UWS or MCS) or of eDoC according to standardized clinical criteria [1].

Within one week from enrolment (T0), the enrolled patients will undergo clinical-functional evaluations, neurophysiological assessments and serum blood sampling at study entry as described in Table 3 for primary, secondary, and exploratory outcomes. After the entry evaluation (T0), patients will perform a total of 25 (5 per week for 5 consecutive weeks) 30 min VEM or TV sessions in the same period of the day. Both interventions (i.e., VEM and TV) are non-invasive, utilize CE-marked medical devices, and are routinely used in neurorehabilitation practice. The devices comply with the EU Medical Device Regulation (MDR 2017/745).

In each daily session, patients will be gradually verticalized (from supine up to a maximum of 90° upright position), using a combined robotic-assisted tilt table with lower limb mobilization, Erigo^®^ (Hocoma, Volketswil, Switzerland), (VEM group) or conventional non-robotic tilt table (TV group). The robotic device combines verticalization with rhythmic controlled passive alternating loading and unloading of the legs of which protocol (e.g., simple stepping, sinus walking) will be set up depending on the patient’s osteoarticular condition (e.g., range of movement = 45°; pace = 24 steps/min), without functional electric stimulation. Legs stepping movements will be obtained with the rhythmic alternating feet pushing up that will be tailored from complete (100% of assistance) to no (0% of assistance) passive mobilization for each limb according to the patient’s motor and cognitive ability and side effects such as pain (see Appendix A for the list of available tasks). Similarly, patients assigned to the TV group will be gradually verticalized (from supine up to a maximum of 90° upright position), according to the patient’s clinical, motor, and cognitive status, using a traditional tilt table. The verticalization in both arms of the trial, and the stepping frequency, and the percentage of assistance for each leg in VEM, will be tailored based on patients’ vital parameters, tolerance to verticalization, and pain (see outcome measures below). VEM and TV training will be performed with the supervision of a physiotherapist and a physician will be always available in case of emergency. In the robotic arm, blood pressure drops were managed—before tilt reversal—by increasing stepping frequency or encouraging active pedaling in conscious patients. These strategies were not available in the traditional tilt-table group, where blood pressure (BP) recovery relied solely on repositioning.

During each VEM or TV session, BP, heart rate (HR), and oxygen saturation will be monitored throughout the entire session to prevent complications associated with orthostatism (e.g., hypotension and syncope). In unconscious patients, these vital parameters will be continuously monitored. In conscious patients, they will be recorded at each verticalization step or whenever symptoms or clinical signs potentially indicative of hypotension or cardiac rhythm disturbances occur (e.g., reduced arousal, pallor, nausea). An ad hoc checklist (Adverse Events Report) will be used to record the following possible adverse events (AE), which will also be documented in REDCap: (i) orthostatic hypotension, defined as a sustained drop in systolic BP ≥ 20 mmHg or diastolic BP ≥ 10 mmHg within 3 min of tilt angle change, according to the diagnostic criteria established by the American and European Autonomic Societies [20]; (ii) postural tachycardia syndrome, defined as a sustained HR increase ≥ 30 bpm within 10 min of standing, in the absence of orthostatic hypotension [20]; (iii) bradycardia, defined as HR < 50 bpm; (iv) oxygen desaturation < 90%; (v) signs of excessive pain or clinical discomfort, particularly in patients with pDoC (e.g., agitation, self-removal of medical devices, violent or unsafe movements).

To ensure rapid activation of emergency procedures in the event of AE during verticalization, all patients must have a functioning intravenous access to allow prompt pharmacological intervention if needed. Airway suction, oxygen delivery systems, and a crash cart are readily available; physiotherapists are trained in their use. A nurse and a physician are available on the ward for rapid intervention if required. In the event of orthostatic hypotension, the following steps are implemented: (i) immediate return to the previous tilt angle; (ii) if unresolved, return to 0°; (iii) if hypotension persists, the patient is placed in the Trendelenburg position; (iv) if instability continues, pharmacological treatment is initiated under medical supervision via the pre-established IV access.

In addition, we defined a graded framework for AE seriousness and relatedness to guide session management:

- Mild AE: No session suspension required. Symptoms are transient and resolve without intervention (e.g., mild and temporary facial pallor without changes in vital signs);
- Moderate AE: Interruption of a single session is required. Symptoms resolve within the same day (e.g., oxygen desaturation resolving after endotracheal suctioning);
- Severe AE: Permanent discontinuation of treatment is considered in case of repeated or persistent AE unresponsive to intervention (e.g., hypotension not responding to reduction in tilt angle), or based on medical judgment prioritizing patient safety.

AEs will be managed according to the 90/385/EEC and 93/42/EEC regulations.

A concomitant usual care and rehabilitation interventions will continue without restriction for both arms. Regardless of allocation to the VEM or TV group, all patients will carry out the comprehensive rehabilitation program as designed by the participating center for the entire study period. The usual rehabilitation program consists of alternating bed positioning, passive limb mobilization, cognitive training, activities to facilitate recovery of consciousness (e.g., multisensorial stimulation), speech, and swallowing therapy. During VEM in sABI study, clinicians will be asked to exercise caution when prescribing “prohibited” (i.e., not permitted under the study protocol) and “proscribed” medications (i.e., permitted but requiring careful documentation and, when necessary, adjustment in timing or dosage). These medications may compromise safety (prohibited agents associated with orthostatic hypotension), and affect the interpretability of outcomes by influencing cognition, attention, arousal, or consciousness (proscribed medications) [21,22]. A list of contra-indicated medications and/or cautions is reported in the Appendix B. Information on pharmacological treatments will be collected at baseline and at all follow-up time points.

At the end of the fifth week of treatment (T1), and after 1 month from T1 (T2) a clinical–functional and neurophysiological evaluation will be performed. A serum blood sample will be collected at T2 (see Table 3).

Clinical–functional and neurophysiological evaluation will be preferably performed early in the morning, after customary nursing procedures, before the rehabilitation treatment. In case of low vigilance of the patient, the CRS-R arousal facilitation protocol [23] will be administered before the evaluation.

### 2.4. Outcome Measures

Almost all measures will be collected at study entry (T0) and at T1 and T2 to evaluate the (possible) changes in the two follow-ups with respect to T0. Primary, secondary, and exploratory outcomes and related measures are summarized in Table 3.

The clinical–functional evaluation includes the following:

- The Level of Cognitive Functioning (LCF) [24,25], an observer-rated scale (from coma to purposeful appropriate behavior) divided into eight levels that describe the patterns or stages of recovery typically seen after a brain injury.
- The Coma Recovery Scale-Revised (CRS-R) [23,26] is an observer-rated tool designed to assess patients with disorders of consciousness. It evaluates auditory, visual, motor, oromotor/verbal, communication, and arousal functions to help differentiate between vegetative state, minimally conscious state, and emergence from these conditions.
- The Disability Rating Scale (DRS) [27], an observer-rated, 30-point continuous scale that provides quantitative information to document the progress of patients with severe brain injury from coma to community reintegration. It evaluates 8 areas of functioning, organized into 4 categories: (1) consciousness (eye opening, verbal response, motor response); (2) cognitive ability (feeding, toileting, grooming); (3) dependence on others; (4) employability. Each area of functioning is rated on a scale of 0 to either 3, 4, or 5 (maximum score = 30-death, minimum score = 0-person without disability) with the highest scores representing the higher level of disability.
- The Modified Ashworth Scale (MAS) [28], an observer-rated scale used to measure muscle spasticity by assessing resistance during passive soft-tissue stretching. Scores range from 0 (no increase in muscle tone) to 4 (rigid in flexion or extension).
- The Medical Research Council (MRC) scale [29], an observer-rated scale for assessing muscle strength, scoring from 0 (no contraction) to 5 (normal strength) in specific muscle groups.
- The Fondazione Don Gnocchi Clinical Complication Scale (FDG-CCS) [30], an observer-rated tool for recording and grading clinical complications during rehabilitation of patients with sABI, covering medical, nursing, and functional events to support patient monitoring and care planning.
- The modified Barthel Index (mBI) [31], an observer-rated continuous scale for evaluation of ability to perform autonomously personal activities of daily living. It measures physical disability across 10 categories that are scored from 0 to either 5, 10, or 15 (maximum score = 100-independence, minimum score = 0-complete dependence) with the highest scores representing the higher level of independence.
- The System Usability Scale (SUS) [32] is a simple, ten-item 5-point Likert scale giving a global view of subjective assessments of usability, including effectiveness, efficiency, and satisfaction of a device. Higher scores correspond to higher usability.
- The Nociception Coma Scale–Revised (NCS-R) [33], an observer-rated tool to assess pain-related behaviors in patients with disorders of consciousness. It evaluates motor, verbal, and facial responses to noxious stimulation, with higher scores indicating stronger nociceptive responses.
- The Agitated Behaviour Scale (ABS) [34], an observer-rated scale measuring the nature and extent of agitation during recovery from brain injury. It assesses behavioral, emotional, and cognitive components, with higher scores indicating greater agitation severity.

#### 2.4.1. Blood Biomarkers and Sampling Procedure

Three blood biomarkers will be collected, as they have been correlated to neuronal degeneration and plasticity in patients with sABI and pDoC [35]: (i) Brain-Derived Neurotrophic Factor (BDNF), a neurotrophin involved in neurogenesis and synaptic plasticity, which can be upregulated by physical exercise [36]; (ii) Neurofilament Light chain (NF-L), a marker of primary and secondary neurodegeneration in sABI previously linked to long-term axonal degeneration and poor outcome [37]; and (iii) Glial Fibrillary Acid Protein (GFAP), a filament protein that has been found to be related to brain function recovery [38].

For each patient, two blood samples (2 × 6 mL vacutainer tubes) will be collected in the morning following an overnight fast, according to a standardized procedure. Each sample will be labeled with a unique reference ID code. Serum will be obtained by centrifugation at 3000 rpm for 15 min and divided into four 500-microliter aliquots. Serum samples will initially be stored at −20 °C at the enrolling centers for a short period prior to shipment, due to the unavailability of −80 °C storage facilities at the participating clinical sites. Shipments will occur promptly, and upon arrival at the central analysis laboratory, all samples will be immediately transferred to −80 °C for long-term storage. BDNF, NF-L, and GFAP in serum will then be analyzed using the Ella Automated Immunoassay System (Bio-Techne, Minneapolis, MN, USA). Sample collection, storage, and shipment will be performed in accordance with established standard operating procedures, ensuring consistency, traceability, and compliance with best laboratory practices.

#### 2.4.2. EEG Recording and Analysis

EEG recordings will be performed at T0, T1 (post-treatment), and T2, which will evaluate changes in cortical electrophysiological activity that may occur even in the absence of overt clinical improvements in some patients [39]. The neurophysiological assessment will include two consecutive eyes-closed (forced for pDoc patients) EEG recordings:

- “resting-state” EEG (duration 15 min).
- “reactivity” EEG (duration 15–17 min) using the following randomized stimuli (each stimulus twice; interstimulus interval ≥ 1 min):
  - eye opening and (forced) eye closing;
  - proximal noxious stimulation (deep pressure applied to the trapezius muscle on each side);
  - distal noxious stimulation (pressing fingernail beds on each hand);
  - acoustic stimulation (hand clapping);
  - personalized acoustic stimulation (patient’s name);
  - intermittent photic stimulation (5 s trains of flashes at 1-2-8-10-15-18-20-25-40-50-60 Hz)

EEG will be recorded by means of 19 electrodes placed on the patients’ scalp according to the international 10–20 system (Fp1, Fp2, F7, F8, F3, F4, C3, C4, T3, T4, P3, P4, T5, T6, O1, O2, Fz, Cz, Pz) plus electrooculogram and electrocardiogram. Data will be sampled at a minimum of 512 Hzand with hardware LP-HP filters open; a notch filter will be used to eliminate frequencies around 50 Hz for online visualization. EEG recordings will take place with the patient sitting on her/his wheelchair, in morning time after customary nursing procedures and (at least) 10 h after administration of drugs acting on the central nervous system, such as myorelaxants and sedative drugs (e.g., benzodiazepines) to optimize vigilance.

The EEG will be analyzed by means of both qualitative (i.e., visual analysis of EEG background activity and reactivity) and quantitative (e.g., power spectrum, microstate, and connectivity analysis) procedures. Specifically for the qualitative analysis, EEG background activity will be classified into five severity categories, according to recently proposed criteria for patients with sABI and the standardized classification of EEG features [40,41].

### 2.5. Data Collection and Management

Patients’ data will be collected and stored in a dedicated paper-based case report form at each participating center. Patients’ data will then be entered in pseudonymized form by each participating center in a centralized computer database on REDCap. This platform complies with the Health Insurance Portability and Accountability Act in compliance with security measures and will therefore only contain data that has already been pseudonymized. Indeed, the personal data of each patient will be de-identified and replaced by an alphanumeric ID code in an ‘association key’ document accessible exclusively to the enrolling center in a password-protected Excel file and then entered into REDCap. The alphanumeric codes will be constructed according to the following order: numeric ID of the center responsible for the case; number of the recruited patient. Similarly, the patients’ EEG raw data will be transmitted in a pseudonymized European Data Format to the coordinating center via REDCap.

Each center will be granted to review only data of their own patients and will be blinded to data entered by other centers. The completeness of the data will be checked regularly by the team of the coordinating center, who will have the possibility to review data of all participating centers. If any missing data or inconsistencies are detected by the coordinating center, the participating center in question will be encouraged to check it thoroughly. Patients’ data collected and rendered pseudonymized by the participating centers will be analyzed in aggregate by the coordinating center.

Monthly call meetings between all participating centers and the Clinical Trial Unit of the coordinating center will be planned, for ensuring the appropriateness of the patients’ enrollment, of the informed consent collection, and of data privacy management, of quality of data acquisition, and of any reported adverse events.

### 2.6. Statistical Plan

All analyses will be conducted by blinded statisticians. Frequencies (percentages) or mean ± standard deviation will be used for descriptive statistics regarding socio-demographic and clinical variables, as well as for the level of satisfaction with the experimental intervention and the occurrence of side effects.

Given that most outcomes are ordinal or exhibit skewed distributions, we will employ linear or generalized linear mixed-effects models (LMM/GLMM) with appropriate link functions. Ordinal outcomes will be analyzed using cumulative logit mixed-effects models, with random intercepts for patients and for enrolling centers. Continuous outcomes approximately normally distributed will be analyzed with LMM, while skewed continuous outcomes will be modeled with appropriate link functions (e.g., log). All models will adjust for baseline values of the outcome (ANCOVA framework) and include time (T0, T1, T2) and group (VEM, TV) as fixed effects, plus their interaction. Model formulas were defined a priori. The following is an example:

logit[P(Y_ij_ ≤ k)] = β_0_ + β_1_Group_i_ + β_2_Time_j_ + β_3_Baseline_i_ + β_4_(Group_i_ × Time_j_) + u_patient(i)_ + u_center(i)_

where u_patient(i)_ and u_center(i)_ are random intercepts for patient and center, respectively.

All analyses will be performed with a two-sided significance level of 0.05. Adjustment for multiple comparisons will be made using Benjamini–Hochberg false discovery rate correction. Statistical analyses will be conducted using R (version 4.4.3; R Foundation for Statistical Computing, Vienna, Austria) or equivalent statistical software. Interim analyses are planned at half of the expected sample for safety outcomes only (i.e., AEs).

Patients violating the VEM-sABI protocol will be excluded from the VEM or TV treatment, but the last clinical observation will be considered for statistical analysis, according to an intention-to-treat approach and a Missing at Random (MAR) assumption inherent to the mixed-model framework. To explore the robustness of the results in scenarios where data may be Missing Not At Random (MNAR), we will conduct pre-specified sensitivity analyses using multiple imputation and, where appropriate, alternative approaches such as joint modeling or delta-adjusted pattern-mixture models.

Protocol violations include: ex-novo administration or change in dose of proscribed drugs (see list in Appendix B), occurrence of severe AE, transfer to acute wards due to severe medical complications, futility of the intervention (as the patient regains the ability to maintain a 90° upright position for ≥15 min without signs of hemodynamic instability on two consecutive days, combined with functional trunk and lower limb control as reflected by a score of ≥3 in the mBI sub-items “Ambulation”, i.e., constant presence of one or more assistants is required during ambulation, and ≥5 in the sub-item “Stair climbing”, i.e., the patient is able to ascend/descend but is unable to carry walking aids and needs supervision and assistance), missing two consecutive sessions or more than five total sessions or request from patients or their legal representative to withdraw from the study.

## 3. Discussion

Patients with sABI are characterized by high clinical complexity, severe cognitive motor disabilities, and an increased risk of developing clinical complications [42,43,44]. An appropriate and skilled comprehensive rehabilitation intervention can significantly impact clinical outcomes and improve the quality of life of patients and their caregivers [1,2,45,46,47]. An early verticalization can promote patients’ recovery and prevent cognitive-motor disabilities and clinical sequelae [3,4,5]. The present multicenter VEM-sABI study will provide new insights into the still-debated efficacy and usability of a device considered safer for verticalization of patients with sABI because it combines robotic verticalization with lower limb mobilization [12]. The study aims to achieve several outcomes. Firstly, it is expected to enroll a large and homogeneous cohort of patients with sABI admitted to post-acute rehabilitation settings. This goal could minimize the methodological bias of the available literature on this field, which most often involved small and heterogeneous patient cohorts (e.g., chronic patients with sequelae of sABI) [5,8,12,13,14]. In addition, we planned to specifically address this topic in post-acute rehabilitation, where the likelihood of patient clinical improvements due to neuroplasticity [18] and the occurrence of clinical complications are higher [42,43,44]. Moreover, we expect that the two cohorts of patients allocated in the experimental and control arm will not differ in terms of demographic and anamnestic characteristics, to avoid biases in evaluating efficacy and usability of the two treatments. Conversely, differences in clinical characteristics and cognitive functions between patients with pDoC and those eDoC are expected. For this reason, targeted clinical and neurophysiological measures will be adopted to detect specific changes in outcome as a function of clinical diagnosis and to identify patients’ profiles with high probability to respond to both (or at least one of the two) treatments. Additionally, we expect the following study outcomes from the data analysis: (i) an improvement of cognitive levels, disability, and motor function at the end of the treatment in both arms, with a significant recovery in the VEM group, in accordance with previous studies [14]; (ii) a more consistent improvement of lower limb strength, and higher reduction in spasticity in the VEM group because of combined controlled mobilization in the VEM device; (iii) lower occurrence of side effects (e.g., orthostatic hypotension) and related dropouts in the VEM arm; (iv) higher occurrence of discontinuation of verticalization due to “treatment futility” in the eDoC patients, as they are more susceptible to early regain vertical posture and gait. A further analysis of motivations of dropouts will identify clinical profiles of patients who could better match the robotic (VEM) or the traditional tilt table treatment (TV); (v) a reduction in clinical complications occurrence in both groups, based on the potential impact of verticalization on patients overall clinical stabilization [3,4]. Because most participating centers are not familiar with this robotic technology in rehabilitation of patients with sABI, we expect to see higher therapist’s compliance for traditional tilt table (i.e., higher SUS score), especially in the early stages of the study. A preliminary structured 3-day training provided by the robotic device manufacturer, on the appropriate use of the robotic device, including real-life testing on at least two different patients, will be performed to avoid potential challenges and ensure the homogeneity in the device use. To ensure ongoing protocol fidelity and inter-site consistency, we implemented the following measures: (i) competency checklist for certification to assess each operator’s ability to manage setup, tilt procedures, stepping control, safety monitoring, and patient interaction; (ii) monitoring parameters (stepping frequency, tilt parameters, adverse events). Ongoing technical and clinical support, throughout constant availability via phone calls and monthly video-call meetings, will be further provided by the coordinator to address possible concerns. We also forecast to identify changes in neurophysiological indices (as measured by EEG) and blood-biomarkers of brain plasticity in patients who will present clinical improvements. In addition, we hope that neurophysiology and biomarkers can detect any sub-clinical (para-clinical) changes caused by the two verticalization procedures. Thus, the two outcome measures will be able to provide useful information to identify patients with a high probability of response to verticalization and help clinicians plan the most appropriate and personalized treatment in such a specific population of patients with sABI.

This trial has several potential limitations. Firstly, the mandatory timing of the study, due to the expected expiration of the funded project, will not allow the long-term effect of the treatment to be investigated. At the end of data collection, if deemed feasible, we hope to be able to follow up some patients over the long term after requesting an amendment from the ethics committee. In general, further studies on large cohorts of patients will be needed to assess patients’ progress after discharge from post-acute rehabilitation.

Secondly, patients will receive the usual care in addition to the experimental or control intervention. Given the different centers participating in the trial, this might act as a confounding factor. However, only Italian centers participated in the trial: in Italy, post-acute rehabilitation is regulated by national legislation, and only minor differences may vary from region to region. For example, the time that is dedicated to the daily rehabilitation of each patient is homogeneous across centers. In addition, any critical differences in usual care between the different centers were discussed and ironed out during initial protocol sharing meetings.

Thirdly, here we only focused on the effect of robotic verticalization vs. traditional verticalization on patients’ outcomes, overlooking possible effects on family caregivers’ satisfaction and burden. Due to limited resources, we could not assess this matter here, but we hope to investigate it in future studies.

Fourthly, for the exploratory analysis of serum biomarkers, serum samples will be initially stored at −20 °C due to the unavailability of −80 °C storage facilities at the participating clinical sites. Storage at −80 °C is generally recommended for the long-term preservation of biomarkers such as NF-L and GFAP, but recent evidence supports the long-term stability of these proteins at −20 °C. In particular, a study by Di Muro et al. [48], conducted on bovine samples, demonstrated that NF-L concentrations in both cerebrospinal fluid and serum remained stable at −20 °C and −80 °C over a median storage period of 16.5 months (IQR: 9–20 months). Notably, concentrations were measured using the same automated ELISA platform planned for use in our study, ensuring methodological consistency. Although potential degradation cannot be completely ruled out, these findings support the reliability of our short-term storage approach.

Notwithstanding this limitation, the VEM-sABI trial will provide new insights into the efficacy, safety, and usability of robot-assisted rehabilitation and offer clinicians useful information to take advantage of technological advances to plan an appropriate and tailored rehabilitation in the post-acute phase of such a complex population.

## Figures and Tables

**Table 1:** Schedule of enrolment, interventions, and assessments from the VEM-sABI study. Each week includes 5 intervention sessions of 30 min duration provided on a daily basis.

Time Point/ Measure	Pre-Randomization	Randomization	Post-Randomization						
	Enrolment	T0 (At the Start of Intervention)	Week 1	Week 2	Week 3	Week 4	Week 5	T1 (After the End of the Intervention)	T2 (1 Month After T1)
Informed consent	X								
Eligibility screening	X								
Allocation		X							
Clinical and anamnestic data collection		X							
Interventions:									
VEM			X	X	X	X	X		
TV			X	X	X	X	X		
Outcomes:									
LCF		X						X	X
CRS-R		X						X	X
mBI		X						X	X
DRS		X						X	X
SUS								X	
MAS		X						X	X
MRC		X						X	X
FDG-CCS		X						X	X
ABS			X	X	X	X	X		
NCS-R			X	X	X	X	X		
30 min standard EEG		X						X	X
Blood sampling		X							X
AER			X	X	X	X	X	X	X

X indicate that activities (rows) are performed at specific time points (columns). Abbreviations: ABS = Agitated Behavior Scale; AER = Adverse Events Report; CRS-R = Coma Recovery Scale-Revised; DRS = Disability Rating Scale; EEG = Electroencephalogram; FDG-CCS = Fondazione Don Gnocchi-Clinical Complication Scale; LCF = Levels of Cognitive Functioning; MAS = Modified Ashworth Scale; MRC = Medical Research Council scale; mBI = modified Barthel Index; NCS-R = Nociception Coma Scale-Revised; SUS = System Usability Scale; TV = Traditional Verticalization; VEM = robotic VErticalization and Mobilization.

**Table 2:** Inclusion and exclusion criteria.

Inclusion Criteria	Exclusion Criteria
Age between 18 and 75 years; sABI due to traumatic, vascular, anoxic, or mixed etiology; Stable behavioral/cognitive diagnosis on at least 4 behavioral evaluations in 1 week using the Italian version of the CRS-R in patients with pDoC, or the LCF-based cognitive assessment in patients with eDoC; Time post-injury between 28 days and 6 months [1]; Not recovered upright station; Written informed consent by the patient’s legal representative/primary caregiver.	Severe medical conditions hampering verticalization (e.g., severe hypotension or conditions realizing hemodynamic instability, end stage or severe symptomatic heart failure with reduced ejection fraction, cardiac arrhythmia of new diagnosis or arrhythmic flare, severe hepatic failure, chronic severe lower limb arterio-venous disease, sepsis/septic shock, thrombus venous embolism of new diagnosis, severe autonomic dysreflexia); Severe medical conditions hampering lower limb mobilization (e.g., fractures, heterotopic ossifications); Severe medical conditions impacting EEG activity (e.g., sub-continuous or abundant EEG epileptiform abnormalities); Severe medical conditions influencing consciousness/cognitive status, such as severe hyponatremia or hypoglycemia; Contraindications to the use of Erigo^®^Basic and/or Erigo^®^Pro (Hocoma, Volketswil, Switzerland) as per technical data sheet (see Appendix A); Presence of prohibited drugs (see Appendix B).

Abbreviations: CRS-R = Coma Recovery Scale-Revised; eDoC = emergence from Disorder of Consciousness; EEG = electroencephalography; LCF = Level of Cognitive Functioning; pDoC = prolonged Disorder of Consciousness; sABI = severe Acquired Brain Injury.

**Table 3:** Summary of primary, secondary, and exploratory outcomes and measures.

Outcome	Timing, Measure
Primary outcome: Global cognitive functioning	At T0 and T1: LCF
Secondary outcomes:	
Functional disability Spasticity of lower limbs Muscular strength of lower limbs Functional independence Usability of devices from physiotherapists Adverse events	At T0, T1 and T2: DRS At T0, T1 and at T2: MAS At T0, T1 and T2: MRC. Additional clinical observation of spontaneous or reflex movement to pain stimulation in pDoC patients At T0, T1 and T2: mBI At T1: SUS Each daily session: AER
Exploratory outcomes:	
Level of consciousness in pDoC Burden of clinical complications Neurophysiological indices of brain functionality Blood biomarkers of brain plasticity Changes in cognitive functioning at 1 month after treatment Pain Overall compliance	At T0, T1 and T2: CRS-R At T0, T1 and T2: FDG-CCS At T0, T1 and T2: qualitative (background activity, reactivity) and quantitative (power spectrum, microstates, connectivity) EEG measures At T0 and T2: Serum level of BDNF, NF-L, GFAP At T0 and T2: LCF Each daily session: VAS (for eDoC patients) or NCS-R (for pDoC) Each daily session: ABS

Abbreviations: ABS = Agitated Behavior Scale; AER = Adverse Events Report; BDNF = Brain-Derived Neurotrophic Factor; CRS-R = Coma Recovery Scale Revised; DRS = Disability Rating Scale; eDoC = emerged from DoC; EEG = Electroencephalogram; FDG-CCS = Fondazione Don Gnocchi Clinical Complication Scale; GFAP = Glial Fibrillary Acid Protein; LCF = Levels of Cognitive Functioning; MAS = Modified Ashworth Scale; mBI = modified Barthel Index; MRC = Medical Research Council; NCS-R = Nociception Coma Scale-Revised; NF-L = Neurofilament Light chain; pDoC= prolonged Disorder of Consciousness; SUS = System Usability Scale; VAS = Visual Analogue Scale.

## Data Availability

No new data were created or analyzed in this study. Data sharing is not applicable to this article.

## Supplementary Materials

The following supporting information can be downloaded at: https://www.mdpi.com/article/10.3390/jcm14186628/s1, Supplementary Materials File S1: SPIRIT checklist; TIDieR checklist.

## Abbreviations

The following abbreviations are used in this manuscript:

ABS	Agitated Behavior Scale
AE	Adverse Events
AER	Adverse Events Report
ANCOVA	Analysis of Covariance
BDNF	Brain-Derived Neurotrophic Factor
BP	Blood Pressure
CRS-R	Coma Recovery Scale-Revised
CT	Computerized Tomography
DRS	Disability Rating Scale
EEG	Electroencephalogram
eDoC	emergence from Disorder of Consciousness
FDG-CCS	Fondazione Don Gnocchi-Clinical Complication Scale
GFAP	Glial Fibrillary Acid Protein
HR	Heart Rate
IQR	Inter-Quartile Range
LCF	Levels of Cognitive Functioning
LMM/GLMM	Linear Mixed-effects Models/Generalized Linear Mixed-effects Models
LP-HP	Low Pass-High Pass
MAR	Missing at Random
MAS	Modified Ashworth Scale
MCS	Minimally Conscious State
MNAR	Missing Not At Random
MRC	Medical Research Council scale
mBI	modified Barthel Index
NCS-R	Nociception Coma Scale-Revised
NF-L	Neurofilament Light chain
pDoC	prolonged Disorder of Consciousness
RCT	Randomized Controlled Trial
REDCap	Research Electronic Data Capture
sABI	severe Acquired Brain Injury
SPIRIT	Standard Protocol Items: Recommendations for Interventional Trials
SUS	System Usability Scale
TPI	Time Post-Injury
TV	Traditional Verticalization
VEM	robotic VErticalization and Mobilization
VS/UWS	Vegetative State/Unresponsive Wakefulness Syndrome

## Appendix A

**Table A1:** List of the tasks available on Erigo^®^Basic and Erigo^®^PRO (Hocoma, Volketswil, Switzerland) and employed for the VEM training. The only difference between Erigo^®^Basic and Erigo^®^PRO is the presence of functional electric stimulation and adjustable hip extension/flexion angle in PRO version, which, however, will not be used in the current trial. Technical data could be found here: https://www.hocoma.com/landing-page/erigo-technical-datasheet-landing/?_gl=1*1onln9k*_up*MQ..*_ga*MTgxOTA2NDQwOS4xNzU0OTIxNjE1*_ga_TJ0VMN8KZJ*czE3NTQ5MjE2MTQkbzEkZzEkdDE3NTQ5MjE2MjMkajUxJGwwJGgw (accessed on 1 September 2025).

Original Name	English Name	Description
Sinus	Sinus	Walking with additional high strike phase, step per minute range from 8 to 80
Andatura	Walking	Walking, step per minute range from 8 to 80
Alternato	Alternate	Simple stepping, step per minute range from 8 to 80

## Appendix B

*Prohibited Medications Increasing the Risk of Orthostatic Hypotension*

The following Table A2 enlists all medications that may potentially increase the risk of orthostatic hypotension, particularly in bedridden or immobilized patients. These drugs may compromise the safety of verticalization.

To ensure protocol integrity and patient safety, all such interventions are prohibited during the trial period; patients already receiving any of these interventions at screening should be considered ineligible for enrollment (exclusion criteria). These drugs must not be initiated at any time between T0 and T2; be tapered or discontinued before enrollment, allowing for a minimum washout of 4× the drug’s half-life; be resumed until the study follow-up is complete. The initiation of any listed medication during the study period will be considered a protocol violation. Source for the estimated half-life was the Italian Medicines Agency (AIFA; https://api.aifa.gov.it (accessed on 1 September 2025)).

**Table A2:** Prohibited medications increasing the risk of orthostatic hypotension.

Drug Class	Examples	Estimated Half-Life	Recommended Action
Loop diuretics	Furosemide	1–1.5 h (up to 24 h in renal failure)	Consider holding 12–24 h prior
Beta-blockers	Metoprolol, Atenolol	Metoprolol: 1–9 h Atenolol: Up to 24 h	Reduce dose or hold 24 h prior
Alpha-blockers	Doxazosin, Prazosin	~22 h	Stop 48 h before
ACE inhibitors	Enalapril, Lisinopril	11–24 h	Consider holding 24–48 h before
ARBs	Losartan	up to 24 h	Consider holding 24–48 h before
Tricyclic antidepressants	Amitriptyline, Nortriptyline	25 h	Hold 48–72 h before
Antipsychotics	Risperidone, Quetiapine, Olanzapine	3–30 h (depends on drug)	Review or suspend 48 h before
Dopaminergic agents	Pramipexole, Ropinirole	>6 h	Taper or review
Benzodiazepines/Sedatives	Lorazepam, Diazepam	Lorazepam: 12–16 h; Diazepam: >30 h	Hold 24–72 h before
Nitrates	Nitroglycerin, Isosorbide	1–5 h	Suspend the day before
Opioids	Morphine, Oxycodone	Morphine: 2–4 h; Oxycodone: >12 h	Hold 24–48 h before if possible

Abbreviations: ACE = Angiotensin-Converting Enzyme Inhibitors; ARBs = Angiotensin Receptor Blockers.

*Proscribed Drugs*

*Caution/“record and adjust” medications*

These “Proscribed” medications are all drugs that may influence cognitive status, attention, arousal, or level of consciousness. These pharmacological effects can significantly bias the assessment of cognition and reduce the validity of intervention primary outcomes. These medications not necessarily need to be discontinued before the intervention, but must be carefully documented (“recorded”) in the study records (on REDCap registry), and may require adjustments (“adjusted”) in timing or administration according to clinical condition and safety (e.g., give in the evening after intervention or clinical-EEG assessment instead of morning, temporarily reduce dose, etc.), to avoid interference with primary outcomes (e.g., alertness, cognitive performance). If the patient is under one of these drugs at study entry, the patients are eligible if they keep the same dosage during the study protocol. If the dosage changes, or one of these drugs needs to be started or discontinued, this is considered as a protocol violation.

The following Table A3 provides a comprehensive overview of CNS-active drugs that may negatively impact cognitive performance [22,49]. It includes their expected cognitive effects, approximate half-lives, and recommended timing for possible tapering or discontinuation before the intervention onset. These recommendations are based on pharmacokinetics (typically 4× half-life for elimination of clinical effect) and clinical safety considerations.

Table A4 summarizes both pharmacological and non-pharmacological interventions that have been proposed to enhance recovery of consciousness and cognitive functioning in patients with severe brain injury, as outlined by Edlow et al. [21]. While a limited number of interventions (such as amantadine and transcranial electrical stimulation) are supported by moderate-to-high level evidence from RCTs, the majority remain experimental, supported only by preliminary data (e.g., case reports, early-phase trials).

**Table A3:** Proscribed medications negatively impacting cognitive performance.

Drug Class	Examples	Half-Life (approx.)	Cognitive/CNS Effects	When to Stop Before Cognitive Tasks/Intervention
Z-drugs	Zolpidem	2–3 h	Memory alteration, confusion	12–24 h before
Typical Antipsychotics	Haloperidol	14–26 h	Rigidity, slowed cognition, delirium	≥3–4 days before
Sedating Antidepressants	Mirtazapine, Trazodone	Mirtazapine: 20–40 h; Trazodone: 5–13 h	Sedation, psychomotor slowing	Mirtazapine: 2–3 days; Trazodone: 24–48 h before
Anticholinergics	Oxybutynin, Trihexyphenidyl	2–16 h	Confusion, disorientation	≥2–3 days before
Antihistamines	Diphenhydramine	4–9 h	Sedation, attention deficit	24–48 h before
Antiepileptics	Levetiracetam, Carbamazepine	Levetiracetam: 6–8 h; Carbamazepine: 12–17 h	Slowed processing, dizziness	≥2–4 days before
Parkinson’s Drugs	Levodopa	1–2 h	Hallucinations, confusion	Taper 24–48 h before if possible
Corticosteroids	Dexamethasone, Prednisone	Dexamethasone: 3–4.5 h; Prednisone: 3–6 h	Insomnia, psychosis, delirium (high doses)	Avoid night doses; consider tapering 48 h before
Chemotherapeutics	Methotrexate, Cisplatin	Methotrexate: ~3–10 h Cisplatin: variable	Confusion, memory loss	Not suspendable; monitor symptoms
Barbiturates	Phenobarbital	80–100 h	Deep sedation, cognitive decline	≥7–10 days before
Melatonin (high dose)	Melatonin	20–50 min	Mild sedation, drowsiness	≥6–8 h before

Abbreviations: CNS = Central Nervous System.

**Table A4:** Proscribed intervention supporting cognitive recovery in severe acquired brain injury.

Category	Intervention	Evidence Level
Pharmacological	Amantadine	★★★★☆—Supported by one RCT in subacute traumatic brain injury
	Zolpidem	★★☆☆☆—Case reports and EEG, PET studies; paradoxical responders
	Baclofen, Midazolam	★☆☆☆☆—Theoretical rationale; anecdotal use; limited case data
	Amitriptyline, Desipramine, Protriptyline	★☆☆☆☆—Sparse data; potential cognitive modulation via noradrenergic/cholinergic systems
	Modafinil	★★☆☆☆—Case reports and early-phase trials suggest increased arousal
	Psilocybin	☆☆☆☆☆—Experimental stage; partial agonist of the 5-HT2A serotonergic receptor in the central nervous system.
Neuromodulation	Deep Brain Stimulation	★★☆☆☆—Positive results in small case series; no large RCTs
	Transcranial Electrical Stimulation	★★★☆☆—Some positive RCTs (30–50% response in MCS patients)
	Transcranial Magnetic Stimulation	★☆☆☆☆—Inconsistent results; limited RCT evidence
Mechanical	Low-Intensity Focused Ultrasound Pulsation	★★☆☆☆—Early-phase studies and case reports; promising but experimental
Sensory	Auditory, tactile, vestibular stimulation	★★☆☆☆—Some RCTs show behavioral or autonomic improvements
	Personalized music therapy	★★☆☆☆—Small studies and clinical reports suggest potential benefits
Regenerative	Stem cell therapies (IV or intrathecal)	★★☆☆☆—Phase I studies suggest safety and preliminary efficacy
	Neurogenesis, gliogenesis, axonal regrowth	☆☆☆☆☆—Preclinical and conceptual stage

Abbreviations: 5-HT2A = 5-Hydroxytryptamine 2A receptor; EEG = Electroencephalography; MCS = minimally conscious state; PET = Positron Emission Tomography; RCT = randomized control trial; ★★★★☆ = Consistent evidence from RCTs or meta-analyses; ★★★☆☆ = Moderate evidence from small RCTs or cohort data; ★★☆☆☆ = Limited evidence (case series, mechanistic); ★☆☆☆☆ = Anecdotal or theoretical only; ☆☆☆☆☆ = Experimental/preclinical.
